# Predicting the Effect of Single and Multiple Mutations on Protein Structural Stability

**DOI:** 10.3390/molecules23020251

**Published:** 2018-01-27

**Authors:** Ramin Dehghanpoor, Evan Ricks, Katie Hursh, Sarah Gunderson, Roshanak Farhoodi, Nurit Haspel, Brian Hutchinson, Filip Jagodzinski

**Affiliations:** 1 Department of Computer Science, University of Massachusetts Boston, Boston, MA 02125, USA; ramin.dehghanpoor001@umb.edu (R.D.); rfarhoodi@gmail.com (R.F.); nurit.haspel@umb.edu (N.H.); 2 Department of Computer Science, Western Washington University, Bellingham, WA 98225, USA; rickse2@wwu.edu (E.R.); hurshk@wwu.edu (K.H.); gunders7@wwu.edu (S.G.); Brian.Hutchinson@wwu.edu (B.H.); 3 Computing and Analytics Division, Pacific Northwest National Laboratory; Richland, WA 99354, USA

**Keywords:** machine learning, protein mutational study, SVR, RF, DNN, rigidity analysis

## Abstract

Predicting how a point mutation alters a protein’s stability can guide pharmaceutical drug design initiatives which aim to counter the effects of serious diseases. Conducting mutagenesis studies in physical proteins can give insights about the effects of amino acid substitutions, but such wet-lab work is prohibitive due to the time as well as financial resources needed to assess the effect of even a single amino acid substitution. Computational methods for predicting the effects of a mutation on a protein structure can complement wet-lab work, and varying approaches are available with promising accuracy rates. In this work we compare and assess the utility of several machine learning methods and their ability to predict the effects of single and double mutations. We in silico generate mutant protein structures, and compute several rigidity metrics for each of them. We use these as features for our Support Vector Regression (SVR), Random Forest (RF), and Deep Neural Network (DNN) methods. We validate the predictions of our in silico mutations against experimental ΔΔG stability data, and attain Pearson Correlation values upwards of 0.71 for single mutations, and 0.81 for double mutations. We perform ablation studies to assess which features contribute most to a model’s success, and also introduce a voting scheme to synthesize a single prediction from the individual predictions of the three models.

## 1. Introduction

The amino acid sequence of a protein determines its structure and as a result, its function. Even a single amino acid substitution can alter a protein’s shape, which can be the cause of debilitating diseases. One notable example includes Fabry disease, a disorder that causes cardiac and kidney complications, which is caused by mutations of α-galactosidase [1].

Wet-lab experiments are used to engineer a protein with a specific mutation, and the mutated protein can be used to infer the effect of that amino acid substitution. The wild type and mutant proteins can be denatured to determine their relative unfolding rates, from which the free energy of unfolding (ΔΔG) can be calculated; it is an indicator of whether a particular mutation is stabilizing or destabilizing, and to what degree. Existing experimental data about mutation studies in proteins is available in the ProTherm database [2].

Unfortunately, conducting mutagenesis experiments on physical proteins is expensive and time consuming; thus, experimental data about the effects of mutations is limited. Therefore, computational methods can be helpful in estimating the effects of a mutation on a protein structure, and several methods have been developed in the past, with various degrees of success.

In the following section, we survey the existing experimental and computational work for predicting the effects of amino acid substitutions on the structure and stability of a protein.

### 1.1. Related Work

#### 1.1.1. Experimental Mutagenesis

Wet-lab experiments provide the gold standard for directly measuring the effects of mutations on a protein’s stability, measured by the ΔΔG of the mutant with respect to the wild type. Matthews et al. have studied many mutants of Lysozyme from Bacteriophage T4 [3,4,5,6,7,8]. It was found that residues with high mobility or high solvent accessibility are much less susceptible to destabilizing substitutions. The downside of experimental studies is that they are time consuming and expensive. Moreover, some mutations are so destabilizing that the mutant protein cannot be crystallized at all. Thus, only a small fraction of all possible mutations can be experimentally studied.

#### 1.1.2. Computational Approaches

Many computational methods have been developed over the years to predict the effects of mutations on protein structure and stability. Several of these methods achieved high prediction and accuracy rates in the 70–80% range.

Several methods consider the backbone of a protein fixed, and perform a search for the best side-chain conformation. Many of them use rotamer libraries to search for the best side chain conformation upon an amino acid substitution [9,10,11]. Other studies used heuristic energy measures [12], database driven potentials [13] or Molecular Dynamics simulations [14]. Thus, progress has been made in predicting the effects of mutations on protein stability. However, many such methods rely on computationally intensive energy calculations and are therefore time consuming.

#### 1.1.3. Combinatorial, Rigidity Based Methods

**Figure 1:**
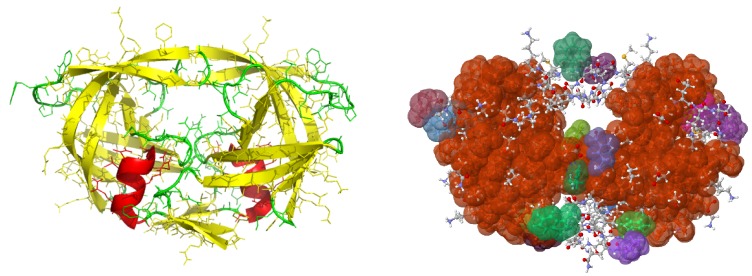
Cartoon (**left**) and Rigidity analysis (**right**) of PDB file 1hvr. Atoms in different rigid clusters are colored by cluster membership. The largest rigid cluster (red-brown) spans both halves of the protein.

Rigidity analysis was first used to explore the effects of mutations involved by calculating a rigid cluster’s configuration entropy value [15]. Later tools for rigidity-based mutation analysis were developed, but the extent of the types of in silico mutations that they could perform were limited. Rigidity Analysis [16,17] is a combinatorial technique for identifying the rigid and flexible regions of biomolecules. Figure 1 depicts the cartoon and rigidity analysis results of Protein Data Bank (PDB) file 1hvr of HIV-1 protease. Rigidity analysis, which identifies rigid clusters of atoms, is distinguished from most other methods by being very fast. It does not rely on homologous protein data, nor on costly all-atom energy calculations. See [17] for a detailed explanation of rigidity analysis.

In our initial previous work we used rigidity analysis to probe how an in silico mutation to glycine destabilizes a protein’s structure. We compared the rigidity properties of the wild type structure to the rigidity properties of a mutant that we generated using KINARI-Mutagen [18]. On input of a PDB structure file, KINARI-Mutagen identifies hydrogen bonds and hydrophobic interactions. The stabilizing interactions involving the atoms of the side chain being mutated to Glycine are removed from the protein’s model. This is equivalent to computationally mutating a specific residue to Glycine, the smallest amino acid which has no side chain atoms that form stabilizing bonds. Later, we combined rigidity analysis with evolutionary conservation to find out if the two measures could give us richer, more complete information about important parts of the protein structure [19]. In subsequent work, we found out that even a simple, SVM-based machine learning scheme, gave better predictions [20].

#### 1.1.4. Machine Learning Based Approaches

Machine learning is a branch of artificial intelligence involving algorithms that learn to make predictions from data. Machine learning has been used to predict the effects of mutations and to infer which residues are critical. Cheng et al. [21] used Support Vector Machines to predict with 84% accuracy the sign of the stability change for a protein due to a single-site mutation. It should be mentioned that Cheng used binary classification and not regression as described in our work here. Also, data of amino acid replacements that are tolerated within families of homologous proteins have been used to devise stability scores for predicting the effect of residue substitutions [22], which has been extended and implemented into an online web server [23]. Brender, et al. [24], have developed a scoring function that reasons about protein-protein interfaces. Machine learning methods have also been used to predict methylation sites [25], phosphorylation sites [26] and protein subcellular locations [27]. They used sequence- and residue-level energy potentials in conjunction with a Random Forest (RF) approach to achieve a Pearson correlation coefficient of approximately 80% between predicted and observed binding free-energy changes upon mutations. Jia, et al. [28], have employed a variety of machine learning tools to generate several models based on thermostability data for assessing the effects of single point mutations. They used 798 mutants from 51 different protein structures for which there is ΔΔG data, and attained accuracy rates ranging from 78–85% among support vector machine, random forest, Naive Bayes classifier, *k*-Nearest Neighbor, neural network, and partial least squares approaches, with the Random Forest Approach having the highest accuracy. Li, et al. [29], developed a model based on the Random Forest algorithm for predicting thermostability changes due to amino acid substitutions. In their approach they relied on 41 features, and achieved accuracies of 79.9%, 78.2%, and 78.7% for single, double, and multiple point mutations.

#### 1.1.5. Model Ensembling

A natural way to combine multiple classifiers is a stacked ensemble [30,31,32]. This technique uses the output of many “level 0” machine learning models, all trained on the same task, to train a new, “meta-learning” model. Many stacked ensembles use the super learner [33] or subsemble [34] algorithm to train from level 0 predictions. At the time of this writing, popular machine learning libraries like TensorFlow and Scikit-Learn do not feature built-in stacked ensemble tools. H_2_O.ai was the only tool found by the authors which supports a stacked ensembles [35]. However, it was not used in this work, as H_2_O’s software stack was not compatible with our existing research pipeline.

Instead of ensembling, in this work we frame the outputs of multiple machine learning models as results from different experiments, and seek to synthesize these results using a meta-analysis framework. Contemporary meta-analysis methods assume that data is not labeled, and therefore accuracy metrics like RMSE and C are not included in this literature [36].

### 1.2. Motivations and Contributions

As discussed above, existing wet-lab experimental methods still provide only partial information about the effects of mutations. Computational methods can complement the mutagenesis work done on physical proteins, but many existing methods are time consuming, or alternatively, their accuracy could be improved. There is a need for fast and reliable methods that can efficiently analyze the effects of a mutation of an amino acid on the structure of a protein. As already discussed, machine learning-based methods have been used in the past and they are a promising avenue to explore further.

In this work, we present fast and efficient machine learning and graph theory-based methods for predicting the effect of mutations on a protein structure. Through rigidity analysis, support vector regression, random forests and deep neural networks, we predict the effect of a mutation on the ΔΔG of a protein. We validate our results by using experimental data from the ProTherm database. In addition, in the past, our pipeline was capable of performing rigidity analysis on mutations to Glycine, Alanine or Serine only, and hence our data involving multiple mutations was extremely limited [37]. Our most recent software permits us to in silico mutate every amino acid to any of the other 19 naturally occurring ones [38]. This allows us to conduct prediction experiments, and incorporate into our training and testing of our machine learning models, hundreds of mutation data points for which there exists ProTherm data and which we previously were not able to use. The data set of proteins with two mutations is still smaller than the set of proteins with single mutant information, but it is large enough to give us better confidence in our predictive ability of the effects of multiple mutations.

To assess the contributions of each of our metrics in a model’s prediction performance, we conducted a systematic ablation study. We one-by-one removed features from the input to each model, and tallied the resulting performance.

We also present a voting scheme that unifies the three machine learning-based approaches into a single prediction. In many cases, our voting approach is able to achieve better prediction accuracies than any single machine learning approach. Our methods often achieve very strong performance in predicting the effect of single and multiple point mutations on a protein structure, while being fast enough to run in a few minutes and sometimes seconds.

Several aspects of our work distinguish it from others. Firstly, none of our features in use by our machine learning models require calculating energetics of various biophysical phenomena. Our features are not dependent on hydrogen bond energies, nor van der Waals forces, nor any other force. Our features are strictly structure-based, which is a purposeful design decision to enable near real-time run-times. Secondly, the number of data points that we use is far more than most others have used. With 2072 mutations for which we have experimentally derived data from ProTherm, our dataset far surpasses in size most others, many of which have fewer than 1000. This dataset is far greater than we used in our previous work due to our recent expanded capabilities of generating mutations in silico. Lastly, the majority of our features in use by our models are derived from quick calculations detailing the rigidity properties of mutant, wild type pairs of protein structures. With the exception of our past proof-of-concept work, nobody else has used rigidity metrics on a large scale to assess their use in enhancing models for predicting the effects of mutations.

This paper extends our recent work [39] because it involves the analysis of mutants with multiple amino acid substitutions, and also includes a meta-analysis voting scheme that synthesizes the results of our three models.

## 2. Results

The support vector machine, random forest and deep neural networks models were trained using the training set, and the hyperparameters were tuned to optimize development set performance as described in the Methods section of this paper. After the optimal parameters were identified, in a final round of training, the samples in the training set and development set were combined and used as an expanded training set. We repeated our experiments using the features mentioned below for the six Rigidity distances (see Methods). The prediction accuracy of the models on the testing set were evaluated by two metrics: Root Mean Square Error (RMSE) and the Pearson correlation (R) between the predicted and actual ΔΔG values.

**Figure 2:**
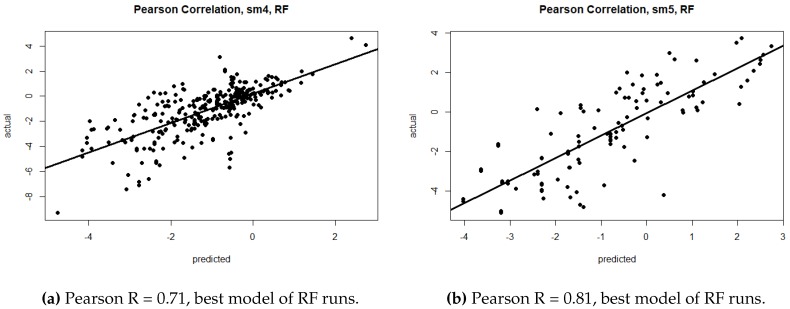
Test set predicted versus actual ΔΔG for single (**a**) and multiple (**b**) mutation data.

**Figure 3:**
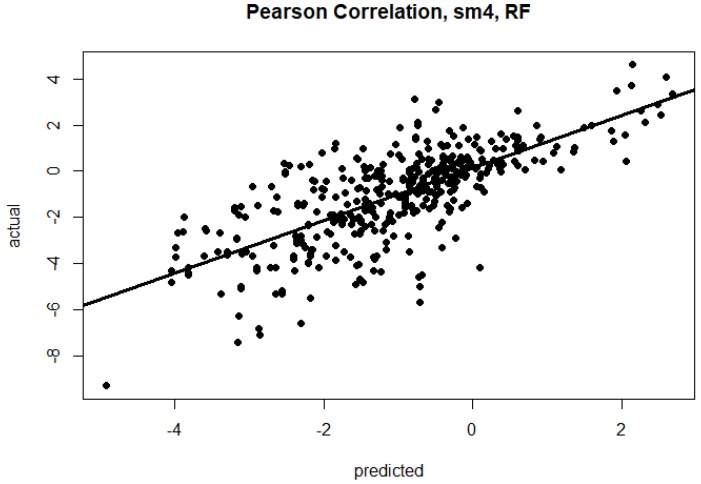
Test set predicted vs. actual ΔΔG, R = 0.73, for best RF model using sm4 rigidity distance metric, for the combined single and double mutations dataset

In the first six numerical columns, Table 1 shows the results when we train and evaluate on single mutation data, and when we trained and evaluated on double mutation data. We also conducted experiments in which the models were trained on the union of the single and double mutation data (see Methods). Table 1 also shows the results for the combined data sets. The plots of calculated vs. predicted value are shown in Figure 2 and Figure 3. As shown in these tables, the RF method gave the best results by producing lower RMSE and a higher correlation. Overall, the results of the double mutant data is better than the single and combined data. The highest performing combination is highlighted in bold font. All rigidity scores tie for the highest correlation (0.71) for the single mutation dataset. The highest correlation for double mutation (0.81) is observed when the rigidity distance score sm1, sm2 or sm5 is used. Finally, RF generated the highest correlation (0.73) for combined mutation when sm4 is used. It should be mentioned that the difference between rigidity scores was rather small, and that we did not observe very large differences between the rigidity scores within each method. This is consistent with our previous studies [37,39].

Because the DNNs exhibited highly varying performance depending on the random weight initialization, we consider a small ensemble approach, in which we trained five DNNs using the same hyperparameters but different random weight initializations, and report their average performance in Table 1. Among the methods, the DNN performs the worst. DNNs are best suited for problems with very large training sets. To better understand the poor generalization of the DNN, we contrast training set and test set performance in Table 2. It shows that the DNNs also generally perform worse on the training set, suggesting that the hyper-parameter tuning process selected a relatively underfit, rather than overfit, model as the one with best generalization. In contrast, the RF is able to fit the training set quite tightly (with training set R up to 0.97) while still generalizing well to the test set. Semi-supervised methods, including pre-training strategies, may be needed to effectively employ DNNs to this problem.

### 2.1. Voting

As described in the Methods section, we explored the benefits of model voting schemes in two scenarios: when only two similarly-performing models generated predictions, and when all three models generated predictions.

#### 2.1.1. Two Model Voting

**Figure 4:**
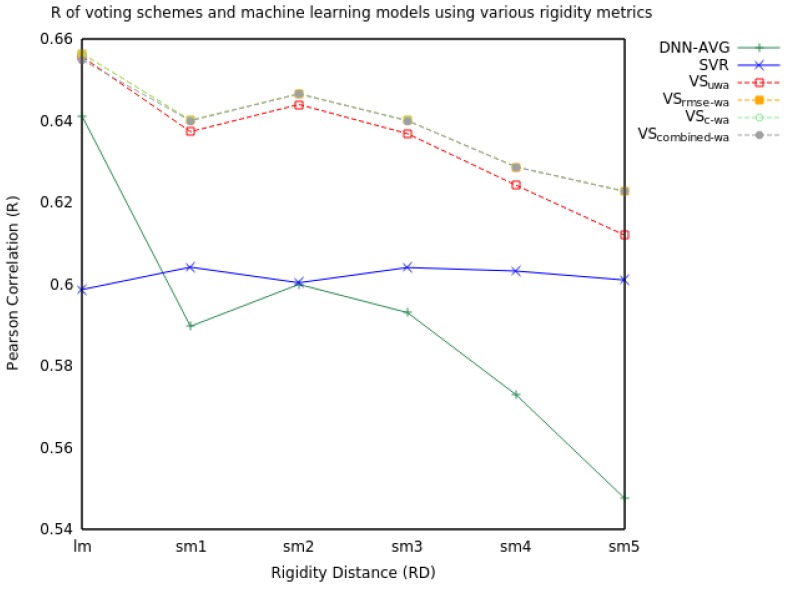
Pearson Correlation (R) values for voting schemes using SVR and DNN predictions for a single mutation using various rigidity distances.

Table 3 shows that voting can improve performance when used on two models that are evenly matched in terms of model performance. Because the DNN results in Table 1 are average RMSE and R values over five distinct models, we perform voting here instead with a single ensemble DNN, whose predictions are the average of the five random models. The Pearson correlation for various rigidity distances is also shown in Figure 4. We refer to this ensemble as “DNN-AVG.” Note that the R and RMSE for the ensemble are slightly better overall than the average of the R and RMSE for the individual models. As shown, all voting schemes yielded higher R and lower RMSE values than the SVR and DNN-AVG models alone.

#### 2.1.2. Three Model Voting

**Figure 5:**
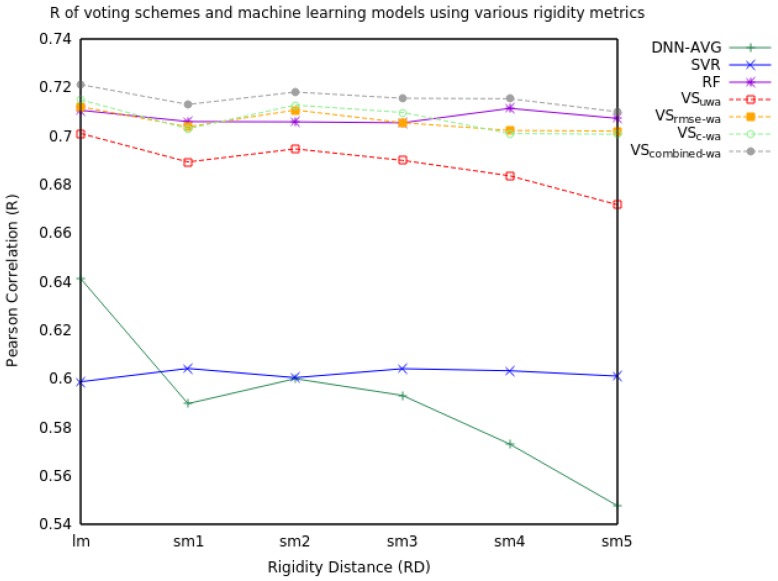
Pearson Correlation (R) values for machine learning models and voting schemes with information from SVR, DNN, and RF predictions for a single mutation using various rigidity distances.

When we extend voting to include all three models, the voting schemes marginally increased performance in RMSE and R (see Table 4 and Figure 5). The VS_combined-wa_ model consistently improved performance over every RD metric.

Future work to increase the voting sophistication has the potential to improve upon this result.

### 2.2. Feature Ablation Study

Although we conducted an ablation study for all models, we report here the ablation study results for the highest scoring RF model. This study was performed by removing one feature at a time, and rerunning the model with the feature removed for each dataset. Via this systematic approach we were able to measure how each feature affects the correlation and RMSE for each Rigidity Distance metric of the data sets (single, double, combined). For single mutations (Table 5), we found that the SASA was consistently the feature that contributed the most to the performance of our method in all cases. Other highly contributing features were the temperature, residue type, pH and some of the rigid cluster fractions, mostly those of smaller cluster sizes. For our double mutation dataset, the ablation study (Table 6) revealed that SASA was the most discriminative feature, but that other features, including cluster fractions, and mutant type, were often important, too. For the combined single and multiple mutations, the ablation study on the RF model (Table 7) reveals that once again SASA is the most important feature, with temperate, and cluster fractions also contributing to the model’s performance.

## 3. Discussion

To assess the utility of our three models in predicting the values of ΔΔG due to point mutations, we compared the Pearson Correlation Coefficients of our Random Forest model (our highest scoring average) against equivalent coefficients for 12 other approaches that we found in the literature [28]. Our Pearson Correlation Coefficient value of 0.71 (combined single and double mutations) would rank our RF approach 2nd of 12, tying with ProMaya, and eclipsed only by ELASPIC having attained higher correlation coefficient value of 0.73, respectively. Understandably, any such comparison must be taken with caution, for example due to different data set sizes, different cross validation approaches, as well as data preprocessing. Although for this work we focused on a regression model rather than attempting a binary classification of the data, it is not uncommon in the literature for binary classification models to exclude neutral (0 ± 0.5 ΔΔG kCal/mol) mutants. Any such similar pre-processing, which we did not do, might be employed by other methods and models attempting regression analyses, which might ultimately affect a ranking of different approaches.

Another important point worth reiterating is that none of our features were attained via direct calculations of energetic terms arising from changes in a protein’s confirmation due to a mutation. Although we previously indicated that doing so was a conscious effort on our part aiming to minimize costly energy calculations, indeed excluding energy terms might be related to a possible limitation of our approach. Namely, a mutation on a protein structure might induce a destabilizing or stabilizing effect due to reasons that are not structure-based, which our method would then not be able to reason about because our features are all purely structural in nature.

## 4. Materials and Methods

In this section, we provide more details about our feature processing, machine learning approaches, voting strategies and ablation study setup.

### 4.1. Data Preparation

#### 4.1.1. In Silico Mutants

Our previous work [39] describes the production of 2072 structures with single mutations. In this work, we also consider double mutations. Of the ProTherm [2] entries that contained two mutations, ΔΔG, temperature and pH values, we were able to gather 457 entries that had valid and easily parsable mutations. Of those, 74 entries had to be removed from the process of in silico mutation due to ambiguity of what chain the experiment was performed on among the multiple chains that had matching source residues to the ProTherm entry. From the remaining entries we were able to run 220 unique in silico double mutations across 21 proteins. This was done using the in-house program ProMuteHT [38] and off-the-shelf programs—Scwrl 4.0 [40] for small-to-large mutations and NAMD [41] for energy minimization.

#### 4.1.2. Rigidity Distance Scores

The effect of a mutation on the protein’s structural stability can be correlated with its effect on a protein’s rigidity. In our previous work [19,20] we measured the effect of the mutation by recording the change in the size of the Largest Rigid Cluster (LRC) of the mutant versus the wild type. The rationale was that the LRC is an indicator of the protein’s flexibility. We achieved over 77% accuracy in predicting the sign of the change of stability for a single point mutation to Glycine and Alanine. In a more recent work we introduced the concept of Rigidity Distance (RD), which relates the rigidity of the wild type and a mutant [37], and for which clusters of all sizes, and not just the largest rigid cluster, were used. The goal was to refine using the distribution of rigid clusters to identify destabilizing mutations. The motivation is that a mutation that reduces the size of the LRC is more destabilizing than a mutation that affects small rigid clusters only. Consider a hypothetical WT and its mutant such that the WT has an LRC of size 17 and the mutant of size 16, but the mutant has 5 times as many clusters of size 10. The count of clusters of size 10 of the mutant more than offsets the small difference between the WT and mutant’s LRC sizes. Our aim was to proportionally incorporate all rigid cluster sizes in determining the effect of a mutation. We developed three RD metrics, where $WT_{i}$ and $Mut_{i}$ are the counts of clusters of size *i* in the WT and Mutant:

$$dm:\sum_{i=1}^{i=LRC}i\times \left[WT_{i}-Mut_{i}\right]$$

$$lm:\sum_{i=1}^{i=LRC}i\times linearWeight_{i}\times \left[WT_{i}-Mut_{i}\right]$$

$$sm:\sum_{i=1}^{i=LRC}i\times sigmoidWeight_{i}\times \left[WT_{i}-Mut_{i}\right]$$

For dm, the number of atoms in a cluster is multiplied by the difference of the count of those clusters in the WT and mutant. Effectively an atom in a large rigid cluster contributes equally to the RD metric as an atom in a small rigid cluster. We did not use this measure for the current study.

For lm, each atom in a cluster of size *i* is scaled by a linear factor based on the LRC. The scale factor is calculated from the equation of the line between the smallest and larger rigid clusters. For example for a protein that has two rigid clusters of size 4, and one rigid cluster of size 22, the equation of the line between the smallest and largest cluster has a rise of 1 (normalized), and run of 22 − 4 = 18. In that case the equation used to calculate the weight for a cluster of size *i* is:

$$linearWeight_{i}=\left(\frac{y_{2}-y_{1}}{x_{2}-x_{1}}\right)\times i+4=\frac{1}{18}\times i+4$$

**Figure 6:**
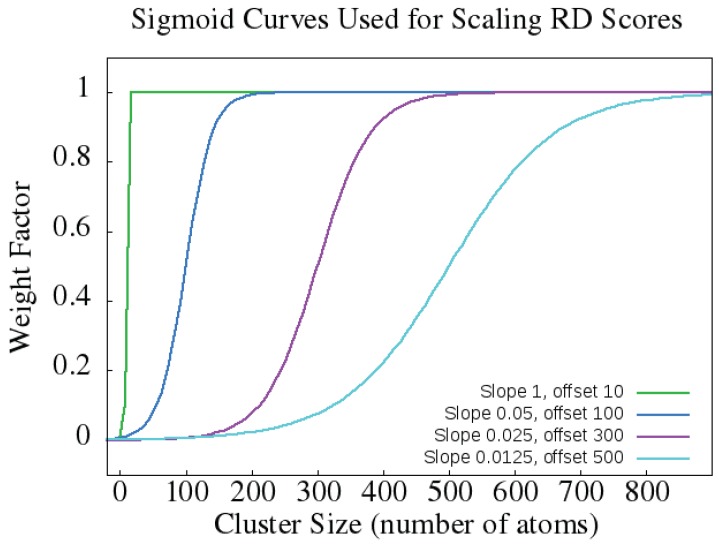
Sigmoid functions for scaling RD metric values. The green sigmoid acts much like a step function and rigid clusters made up of 10 or fewer atoms are weighted by a factor of 0. Using the violet sigmoid, atoms in a cluster size up to 200 atoms would be assigned a near 0 weight, atoms in clusters of size 200–300 would be weighed by 0.1–0.8, and atoms in clusters of 300+ atoms would be weighted by 0.8 or more. Reproduced from [37].

For sm, each cluster size is weighed by a factor according to a sigmoid function. This permits us to weigh the largest cluster sizes proportionally much more than smaller ones. The cutoff point at which cluster sizes contribute significantly to the RD score is determined by the parameters of the sigmoid function. The sigmoid functions that we evaluated for use in the RD metric are shown in Figure 6 (reproduced from our previous work [37]). We found that when rigid cluster sizes were scaled using the green sigmoid function that the sm score correlated best with ΔΔG values. We designate the RD metric that uses the green sigmoid function sm1 hereafter. We also refer to the metric that relied on the violet (slope 0.025, offset 300) sigmoid as sm2.

#### 4.1.3. Feature Extraction

From the ProTherm data and our rigidity calculations, we derived the following features:

- Solvent accessible surface area (SASA): 2 real-valued features (4 in double mutations) indicating how exposed to the surface a residue is (both absolute and percentage).
- Secondary Structure: 4 binary features (8 for double mutations) indicating whether each mutation is part of a sheet, coil, turn or helix.
- Temperature and pH at which the experiment for calculating ddG was performed.
- Rigidity distances (RD): one of lm, sm1, sm2, sm3, sm4, and sm5 (see above and [37]).
- Rigid Cluster Fraction: 48 features giving the fraction of atoms in the WT and MUT that belong to rigid clusters of size 2, 3, ... , 20, 21–30, 31–50, 51–100, 101–1000, and 1001+, respectively.
- Residue type: 8 categorical features (16 in double mutants) indicating whether the mut1wt, mut1target (in double mutants also mut2wt, and mut2target) are Charged (D, E, K, R), Polar (N, Q, S, T), Aromatic (F, H, W, Y), or Hydrophobic (A, C, G, I, L, M, P, V).

For the combined data set which included both single and double mutants, all the categorical and numerical features concerning mut2 were set to 0 for the single mutations, in order to make the number of features equivalent. A binary feature indicating single vs double mutation was also included. While augmenting or whittling this feature set (by feature engineering or feature selection, respectively) may lead to improved performance, we leave this to future work.

#### 4.1.4. Data Split

The data was split into training, development and test sets, for the purposes of training, model selection/tuning and evaluation, respectively. Data was split into these sets under the constraint that each unique wild type mutation combination appeared only in a single set. The statistics for these sets can be found in Table 8.

### 4.2. Machine Learning Methods

We describe the three major machine learning methods employed in this work: support vector regression, random forests and deep neural networks.

#### 4.2.1. SVR

Support Vector Machines (SVMs) are supervised machine learning models that are particularly effective for cases with large feature dimension and small training sets. While most often used for classification, SVMs can be used for regression. This is often referred to as support vector regression (SVR) [42]. These models are trained to minimize regularized empirical risk in order to balance fitting the trends in the data without overfitting noise contained therein. While SVR is by default a linear method, it can model non-linear relationships through the use of a kernel function, which implicitly, non-linearly maps the features into a (potentially infinite dimensional) feature space. Common non-linear kernels include the polynomial, sigmoid and radial basis kernels.

The SVR model that we used in this work for ΔΔG prediction were implemented using the R interface to libsvm [43]. For the SVR we tuned the following parameters by grid search and 10-fold cross validation on the union of the training and development sets: SVM type, kernel and kernel coefficient γ, ϵ. The lowest error was achieved with ϵ equal to 0.1, when the RBF kernel was used and γ was set to 0.015.

#### 4.2.2. RF

Random forests (RFs) are ensemble models that combine the results of numerous decision trees. A decision tree is a simple machine learning model that maps inputs to predictions by traversing a tree structure, in which a question about a feature is asked at each node. It can be used for classification or regression. The trees are learned by applying splitting criteria at each node, such as information gain or Gini impurity index. A random forest ensembles many decision trees that are trained on multiple random sub-samples of the training set and then use averaging to improve the accuracy of the prediction and reduce model variance [44]. Each tree is trained on a set sampled from the full training set, generally with replacement, equal in size to the full set.

The random forest model we used is implemented by the randomForest library in the R programming language [45]. We tuned our random forest model using 10-fold cross-validation on the union of the training and development sets over a range of hyper-parameters. For the resampling method, we used bagging. The best model was achieved when the number of trees is set to 500 and the number of variables tried at each split is set to 22.

#### 4.2.3. DNN

Deep Neural Networks (DNNs) are supervised machine learning models that are composed from a sequence of parametric, differentiable “layers” and trained end-to-end. A shallow neural network, sometimes known as a multilayer perceptron or artificial neural network, has only two parametric layers (a “hidden” layer and an output layer). Mathematically, the output *y* is produced as followed:

(1) $$\begin{array}{ccc} y & = & W_{\left(L\right)}^{T}h_{\left(L\right)}+b_{\left(L\right)} \end{array}$$

(2) $$\begin{array}{ccc} h_{\left(\ell \right)} & = & g\left(W_{\left(\ell \right)}^{T}h_{\left(\ell -1\right)}+b_{\left(\ell \right)}\right)\mathrm{for}i=1,\cdots ,L-1, \end{array}$$

where the input *x* is written as $h_{\left(0\right)}$. There are *L* parameter matrices, *W*, and *L* bias vectors, *b*. The function *g* is known as an activation function and must be non-linear for the overall model to be non-linear. In this work we consider g(z)=tanh(z) and g(z)=ReLU(z)=max(0,z). The model parameters are learned using minibatch stochastic gradient descent to minimize the average training set squared error (MSE). We implemented the DNN using the open-source machine learning library TensorFlow [46].

We separately tuned a set of hyper-parameters for each rigidity metric and data set (single, multiple, and combined single and multiple mutations) using random search on the development set. The hyper-parameters include the number of hidden layers (ranging from 1 to 4), number of hidden units per layer (ranging from 10 to 100), learning rate used in training (ranging from 0.001 to 0.1), the range over which initial weights were uniformly drawn (U(−ϵ,ϵ) for ϵ from 0.001 to 0.1), the number of data points used for each gradient calculation (i.e., minibatch size, in the set {8, 16, 32, 64, 128, 256, 512}), the activation function (in the set {tanh, ReLU}), and the optimizer used for backpropagation (between vanilla stochastic minibatch gradient descent and adam [47]).

### 4.3. Voting Schemes

With three different machine learning models trained to predict ΔΔG values, we seek to combine the strengths of these algorithms to make an improved prediction. In order to leverage the additional information encapsulated in RMSE and R scores, we created our own set of voting methods. Much like a stacked ensemble, these methods use the predictions from multiple machine learning models along with RMSE and R values to “vote” on a winning ΔΔG value which may or may not be present in the input set. To this end, we developed several voting schemes (VS) using single mutation data. We describe each of them below.

VS_uwa_: An unweighted average of all models’ predictions for a given mutation. For *m* models each with an output $h_{i}\left(x\right)\in R$, where i=1,2,⋯,m, our voting prediction is:
  (3) $${\mathrm{VS}}_{\mathrm{uwa}}\left(x\right)=\frac{1}{m}\sum_{i=1}^{m}h_{i}\left(x\right)$$
VS_c-wa_: A weighted average of all models’ predictions for a given mutation, adjusting each model’s prediction based on the strength of its Pearson Correlation Coefficient, R, relative to the model with the best R. Again assume we have a set of models $h_{i}\left(x\right)$ for i=1,2,⋯,m but let $h_{*}\left(x\right)$ denote the output from the best performing model, $c_{i}$ denote the R for model *i* and let $c_{*}$ denote R for the best performing model, then our voting prediction is:
  (4) $${\mathrm{VS}}_{c-\mathrm{wa}}\left(x\right)=\frac{1}{m}\sum_{i=1}^{m}\left(h_{i}\left(x\right)+\left(h_{*}\left(x\right)-h_{i}\left(x\right)\right)\left(1-\frac{c_{i}}{c_{*}}\right)\right)$$
VS_rmse-wa_: A weighted average of all models’ predictions for a given mutation analogous to **VS_c-wa_**, except using RMSE instead of R. In this case, $h_{*}\left(x\right)$ is the prediction of the best model (according to RMSE), $r_{i}$ is the RMSE for model *i* and $r_{*}$ is the RMSE of the best model. Then our voting prediction is:
  (5) $${\mathrm{VS}}_{\mathrm{rmse}-\mathrm{wa}}\left(x\right)=\frac{1}{m}\sum_{i=1}^{m}\left(h_{i}\left(x\right)+\left(h_{*}\left(x\right)-h_{i}\left(x\right)\right)\left(1-\frac{r_{*}}{r_{i}}\right)\right)$$
VS_combined-wa_: A weighted average of all models’ predictions for a given mutation incorporating both the R and RMSE performance. Letting $\gamma_{i}=c_{i}/r_{i}$ and $\gamma_{*}$ denote the best (max) $\gamma_{i}$, our prediction is:
  (6) $${\mathrm{VS}}_{\mathrm{combined}-\mathrm{wa}}\left(x\right)=\frac{1}{m}\sum_{i=1}^{m}\left(h_{i}\left(x\right)+\left(h_{*}\left(x\right)-h_{i}\left(x\right)\right)\left(1-\frac{\gamma_{i}}{\gamma_{*}}\right)\right)$$

Several hyperparameters are introduced to improve these voting scheme baselines in the event that outlier predictions significantly affect a model’s R or RMSE score. In addition to the standard computation of a model’s R and RMSE, we recalculate each model’s RMSE and R after excluding outliers or scaling outliers by a constant, then commence the voting schemes. Outliers are identified by comparing a threshold hyperparameter to the ratio of a prediction’s loss to the model’s RMSE. The best hyperparameter configuration for each scheme was used to generate the results of each voting scheme.

## 5. Conclusions

In this work we present several machine learning-based methods to predict the effects of single and double point mutations on the stability of a protein. In particular, our approach predicts the change to the free energy of unfolding upon mutation (ΔΔG), using a combination of graph-based rigidity analysis and structural features that include solvent accessible surface area (SASA), temperature, pH, secondary structure element and the type of mutated amino acid. We trained and tested our methods on an extensive dataset taken from the ProTherm database, which contains experimental information about single and double point mutations. We also implemented a voting scheme that combines the predictions of our different machine learning methods, resulting in a higher prediction accuracy than any of the methods alone. Our best performing voting scheme gave predictions with a higher R score than any of the three individual machine learning methods. We show that our algorithm, especially the Random Forest (RF)-based predictor, can predict the ΔΔG with high accuracy and low root mean squared error (RMSE).

Our next steps involve exploring more efficient prediction methods, as well as including insertions and deletions. Additionally, since our method is very fast and efficient, taking just a few seconds per protein, we are currently building a server which will allow users to conduct their own predictions using our machine learning prediction scheme.

## Figures and Tables

**Table 1:** Test set results for regression models for single and double mutants, as well as the union of the two (combined). RD = Rigidity Distance. The best results are shown in bold font.

		Single Mutants			Double Mutants			Combined		
RD	Measure	SVR	RF	DNN	SVR	RF	DNN	SVR	RF	DNN
lm	RMSE	1.53	**1.34**	1.60	1.61	1.41	1.74	1.54	1.39	1.71
	R	0.60	0.71	0.58	0.76	0.79	0.66	0.65	0.72	0.52
sm1	RMSE	1.52	1.35	1.60	1.60	1.37	1.64	1.54	1.39	1.80
	R	0.60	0.71	0.57	0.76	**0.81**	0.71	0.65	0.72	0.46
sm2	RMSE	1.53	1.35	1.71	1.61	1.36	1.90	1.54	1.40	1.87
	R	0.60	0.71	0.55	0.76	**0.81**	0.60	0.65	0.72	0.46
sm3	RMSE	1.52	1.35	1.60	1.60	1.38	1.93	1.54	1.39	1.81
	R	0.60	0.71	0.58	0.76	0.80	0.60	0.65	0.72	0.44
sm4	RMSE	1.52	**1.34**	1.57	1.56	1.38	1.83	1.54	1.38	1.77
	R	0.60	0.71	0.57	0.77	0.80	0.64	0.66	0.73	0.55
sm5	RMSE	1.53	1.35	1.70	1.60	1.35	1.89	1.54	1.39	1.74
	R	0.60	0.71	0.52	0.76	**0.81**	0.52	0.65	0.72	0.51
Avg.	RMSE	1.53	1.35	1.63	1.60	1.38	1.82	1.54	1.39	1.78
	R	0.60	0.71	0.56	0.76	0.80	0.62	0.65	0.72	0.49

**Table 2:** Comparing training and test set results for regression models, averaged over the six RD metrics.

Accuracy	Measure	SVR	RF	DNN
Single	Avg. Train RMSE	1.08	0.47	1.04
	Avg. Test RMSE	1.53	1.35	1.67
	Avg. Train R	0.79	0.97	0.79
	Avg. Test R	0.60	0.71	0.56
Double	Avg. Train RMSE	1.08	0.70	1.20
	Avg. Test RMSE	1.60	1.38	1.82
	Avg. Train R	0.83	0.93	0.69
	Avg. Test R	0.76	0.80	0.62
Combined	Avg. Train RMSE	1.11	0.50	1.33
	Avg. Test RMSE	1.52	1.39	1.78
	Avg. Train R	0.79	0.96	0.62
	Avg. Test R	0.65	0.72	0.49

**Table 3:** Test set results for voting schemes for single mutants using SVR and DNN predictions. The voting schemes that showed the best improvement are highlighted in bold fonts.

RD	Measure	SVR	DNN-AVG	VS_uwa_	VS_rmse-wa_	VS_c-wa_	VS_combined-wa_
lm	RMSE	1.53	1.46	**1.43**	**1.43**	**1.43**	1.43
	R	0.60	0.64	**0.66**	**0.66**	**0.66**	0.65
sm1	RMSE	1.52	1.55	1.46	1.46	1.46	1.46
	R	0.60	0.59	0.64	0.64	0.64	0.64
sm2	RMSE	1.53	1.57	1.45	1.45	1.45	1.45
	R	0.60	0.60	0.64	0.65	0.65	0.65
sm3	RMSE	1.52	1.57	1.46	1.46	1.46	1.46
	R	0.60	0.59	0.64	0.64	0.64	0.64
sm4	RMSE	1.52	1.56	1.48	1.48	1.48	1.48
	R	0.60	0.57	0.62	0.63	0.63	0.63
sm5	RMSE	1.53	1.63	1.50	1.49	1.49	1.49
	R	0.60	0.55	0.61	0.62	0.62	0.62

**Table 4:** Test set results for voting schemes for single mutants using SVR, DNN and RF predictions. The best results are highlighted in bold fonts.

RD	Measure	DNN-AVG	SVR	RF	VS_uwa_	VS_rmse-wa_	VS_c-wa_	VS_combined-wa_
lm	RMSE	1.46	1.53	1.34	1.37	1.35	1.35	**1.33**
	R	0.64	0.60	0.71	0.70	0.71	0.71	**0.72**
sm1	RMSE	1.55	1.52	1.35	1.39	1.37	1.37	1.34
	R	0.59	0.60	0.71	0.69	0.70	0.70	0.71
sm2	RMSE	1.57	1.53	1.35	1.37	1.35	1.35	**1.33**
	R	0.60	0.60	0.71	0.69	0.71	0.71	**0.72**
sm3	RMSE	1.56	1.52	1.35	1.38	1.36	1.36	1.34
	R	0.59	0.60	0.71	0.69	0.70	0.71	0.72
sm4	RMSE	1.56	1.52	1.34	1.40	1.37	1.38	1.34
	R	0.57	0.60	0.71	0.68	0.70	0.70	0.72
sm5	RMSE	1.63	1.53	1.35	1.41	1.37	1.37	1.35
	R	0.55	0.60	0.71	0.67	0.70	0.70	0.71

**Table 5:** Test set RF ablation results on single mutations.

	Accuracy Measure	Feature 1	Feature 2	Feature 3	No Ablation
**lm**		**SASA**	**temp**	**type**	
	RMSE	1.48	1.39	1.38	1.34
	R	0.63	0.68	0.69	0.71
**sm1**		**SASA**	**temp**	**type**	
	RMSE	1.50	1.39	1.38	1.35
	R	0.62	0.66	0.69	0.71
**sm2**		**SASA**	**temp**	**type**	
	RMSE	1.50	1.39	1.38	1.35
	R	0.62	0.68	0.69	0.71
**sm3**		**SASA**	**temp**	**type**	
	RMSE	1.49	1.38	1.37	1.36
	R	0.62	0.68	0.69	0.70
**sm4**		**SASA**	**temp**	**type**	
	RMSE	1.49	1.38	1.38	1.35
	R	0.63	0.69	0.69	0.71
**sm5**		**SASA**	**temp**	**type**	
	RMSE	1.50	1.40	1.38	1.36
	R	0.62	0.68	0.69	0.70

**Table 6:** Test set RF ablation results on double mutations.

	Accuracy Measure	Feature 1	Feature 2	Feature 3	No Ablation
**lm**		**mut2SASA**	**mutClusterFrac16**	**wtClusterFrac1001**	
	RMSE	1.47	1.41	1.40	1.39
	R	0.77	0.79	0.79	0.80
**sm1**		**mut2SASA**	**wtClusterFrac11**		
	RMSE	1.46	1.39		1.39
	R	0.77	0.80		0.80
**sm2**		**mut2SASA**	**wtClusterFrac11**	**mut1target_type**	
	RMSE	1.46	1.39	1.38	1.37
	R	0.77	0.80	0.80	0.81
**sm3**		**mut2SASA**	**ph**	**mutClusterFrac11**	
	RMSE	1.46	1.40	1.38	1.37
	R	0.77	0.80	0.80	0.80
**sm4**		**mut2SASA**	**wtClusterFrac18**	**wtClusterFrac101**	
	RMSE	1.45	1.40	1.40	1.37
	R	0.78	0.79	0.80	0.80
**sm5**		**mut2SASA**	**wtClusterFrac11**	**ph**	
	RMSE	1.47	1.39	1.39	1.36
	R	0.77	0.80	0.80	0.81

**Table 7:** Test set RF ablation results on combined mutations.

	Accuracy Measure	Feature 1	Feature 2	Feature 3	No Ablation
**lm**		**mut1SASA**	**temp**	**mut1type**	
	RMSE	1.47	1.43	1.42	1.40
	R	0.68	0.70	0.70	0.72
**sm1**		**mut1SASA**	**temp**	**mut1type**	
	RMSE	1.48	1.42	1.41	1.39
	R	0.68	0.71	0.71	0.72
**sm2**		**mut1SASA**	**temp**	**mut1type**	
	RMSE	1.48	1.42	1.42	1.39
	R	0.68	0.70	0.71	0.72
**sm3**		**mut1SASA**	**mut1type**	**temp**	
	RMSE	1.48	1.42	1.42	1.39
	R	0.68	0.70	0.71	0.72
**sm4**		**mut1SASA**	**temp**	**mut1type**	
	RMSE	1.47	1.42	1.41	1.39
	R	0.69	0.71	0.71	0.72
**sm5**		**mut1SASA**	**temp**	**mut1type**	
	RMSE	1.48	1.43	1.41	1.40
	R	0.68	0.70	0.71	0.72

**Table 8:** Data set sizes.

Dataset	Training	Development	Test	Total
Single	1488	331	320	2139
Double	147	60	107	314
Combined	1635	391	427	2453

## Abbreviations

The following abbreviations are used in this manuscript:

DNN	Deep Neural Network
SVR	Support Vector Regression
RF	Random Forest
LRC	Largest Rigid Cluster
RD	Rigidity Distance
WT	Wild Type
